# Fascial Nomenclature: Update 2021, Part 1

**DOI:** 10.7759/cureus.13339

**Published:** 2021-02-14

**Authors:** Bruno Bordoni, Allan R Escher, Filippo Tobbi, Antonio Pranzitelli, Luigi Pianese

**Affiliations:** 1 Physical Medicine and Rehabilitation, Foundation Don Carlo Gnocchi, Milan, ITA; 2 Anesthesiology and Pain Medicine, H. Lee Moffitt Cancer Center and Research Institute, Tampa, USA; 3 Osteopathy, Poliambulatorio Medico e Odontoiatrico, Varese, ITA; 4 Osteopathic Medicine, Poliambulatorio "Gemelli" Molise, Termoli, ITA; 5 Physical Medicine and Rehabilitation, 3C+A Health and Rehabilitation, Roma, ITA

**Keywords:** fascia, fascintegrity, osteopathic, fascial system, fascial continuum

## Abstract

The fascial continuum is a topic for which all clinicians and other healthcare professionals come into contact on a daily basis, both consciously and without having the idea that the tissues they deal with can fall within the concept of fascia. The Foundation of Osteopathic Research and Clinical Endorsement (FORCE) organization includes many clinicians and health professionals, as well as researchers in different scientific disciplines. The goal is to dissect some concepts related to daily practice, such as fascial tissue, from a scientific point of view and impartially. Proof of the impartiality of FORCE is the fact that it does not sell any fascial products, no tools, and, above all, all the fascial terminology used has no copyright: research and knowledge are the right of anyone who wishes improvement for the good of the patient. The article aims to review the themes that could add new elements for a broader view of the meaning and nomenclature of the fascial system.

## Introduction and background

In scientific literature (PubMed), the first article appearing with the term fascia dates back to 1814, where a doctor describes a surgery, highlighting the concept of connective tissue or fascia that separates the different muscle groups [1]. Another text from 1824 describes the different layers and fascias of the inguinal area, underlining the difficulty in finding a common name for this anatomical portion [2]. Other scientific texts between the late 1800s and early 1900s discuss the different layers of fascial tissue [3-5]. In the twentieth century, after many publications using the term fascia, some anatomical clarifications on the terminology came out by groups of anatomists and researchers, such as the International Anatomical Nomenclature Committee (1983) and the Federative Committee on Anatomical Terminology (1998) [6]. These last two groups highlight some words like "fascia superficialis", "fascia profunda", comparing the fascial tissue as "sheaths, sheets or other dissectible connective tissue aggregations" [6]. In the twenty-first century, these groups have not deviated from these concepts.

In the new millennium, we can find another group dedicated to the study of fascial tissue, the Fascia Nomenclature Committee (FNC), which in turn derives from the Fascia Research Society (2014) [6]. A 2019 article, under the auspices of the FNC, adds a further distinction between the term fascia and the fascial system; for this organization, the first term is equivalent to the description of the tissue (mesoscopic and microscopic scale), while the fascial system contains the concept of functions of the fascia [6]. For the FNC, "a fascia is a sheath, a sheet, or any other dissectible aggregations of connective tissue that forms beneath the skin to attach, enclose, and separate muscles and other internal organs", while the fascial system "... consists of the three‐dimensional continuum of soft, collagen containing, loose and dense fibrous connective tissues that permeate the body" [6].

According to another non-profit organization, the Foundation of Osteopathic Research and Clinical Endorsement (FORCE), the fascial fascia/continuum is defined as: “The fascia is any tissue that contains features capable of responding to mechanical stimuli. The fascial continuum is the result of the evolution of the perfect synergy among different tissues, liquids and solids, capable of supporting, dividing, penetrating, feeding and connecting all the regions of the body, from the epidermis to the bone, involving all its functions and organic structures. This continuum constantly transmits and receives mechanometabolic information that can influence the shape and function of the entire body. These afferent/efferent impulses come from the fascia and the tissues that are not considered as part of the fascia in a biunivocal mode." In this definition, these tissues include "epidermis, dermis, fat, blood, lymph, blood, and lymphatic vessels, tissue covering the nervous filaments (endoneurium, perineurium, epineurium), voluntary striated muscle fibers and the tissue covering and permeating it (epimysium, perimysium, endomysium), ligaments, tendons, aponeurosis, cartilage, bones, meninges, and tongue” [7,8].

What is the scientific basis for defining a tissue (made up of different cells)? Who are the figures who should have an opinion on the same tissue? Trying to answer these two questions, the article also reviews current and updated scientific information to find a more appropriate definition of fascial tissue, underlining its clinical importance and therapeutic potential.

## Review

Fascial tissue embryology: meninges

Going along a taxonomic scale to understand the concept of holobiont is like giving a name to a hologenome without understanding how all the hologenomes interact with each other, within the holobiont itself; that is, giving names to tissues without looking at the function as a whole, without studying the origin of the cells that make up the tissue, is like trying to pierce water with a finger [9,10]. To know a tissue and before naming it, it is necessary to know its origin, in order to correctly identify the anatomical area where it resides, its connections and functions; to understand a macroscopic tissue it is necessary to start from the microscopic components [11]. The ontogenesis of what will become the whole fascial tissue involves the mesoderm and the ectoderm, starting from the first weeks of the evolutionary process [11]. The dual phylogeny of the fascial continuum concerns, in particular, the area of the skull and neck. The portion of the cranial dural tissue, considered as fascia by the FORCE and the FNC, which covers the forebrain and caudal mesencephalon and which constitutes the falx cerebri and the falx cerebelli (and spinal cord), has an ectodermal derivation; the meninges that cover the hindbrain and midbrain, as well as some venous sinuses and the portion of the hypoglossal canal, arise from the mesodermal sheet [11].

Who can define, by observing these tissues in continuity, when an area should be considered as a fascia compared to another cranial anatomical area? No one. The meninges play a fundamental role in the development of the nervous system and in the development of the bones of the skull [12]. The meninges influence cranial bone morphogenesis; are a source of different growth factors (belonging to the transforming growth factor beta or TGFbeta family), such as fibroblast growth factor and bone morphogenetic protein (BMP), which are signal substances for target cells, guiding and directing growth and development [12]. During development, the meninges are critical for the formation of the nervous system. They are able to secrete growth factors (chemokines, BMPs, TGFbeta) for neurogenesis, to avoid apoptosis of nerve cells, for the migration and positioning of neurons, and inducing the development of cerebral blood vessels [12]. In adults, they play a fundamental role in the management of mechanical tensions, trying to protect the brain mass and the medulla from traumatic insults, and influencing the repair of nervous tissue thanks to the presence of stem cells; they secrete growth factors to obtain correct homeostasis of nervous function [12,13].

**Figure 1:**
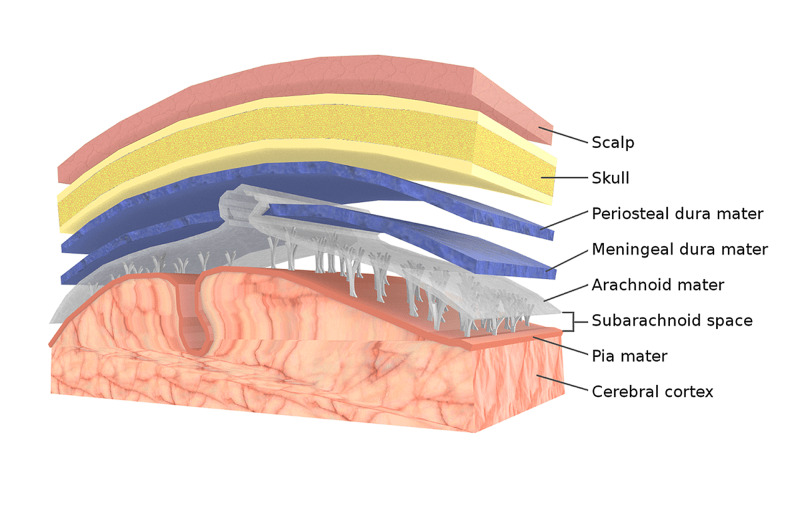
Different layers of the skull, from the skin to the pia mater Source: Part of the Bruno Bordoni image collection.

Looking at the microscopic, we can see some differences. The dura mater or pachymeninge is a collagenous structure, with lamellae of collagen (longitudinal and transverse), fibronectin, and elastin; the dura has viscoelastic properties, with the ability to generate mechanical force or reduce mechanical tension [13]. The arachnoid and the pia mater or leptomeninges are rich in collagen, fibroblasts, laminins, proteoglycans and nidogens or entactins, vimentins [12,13]. The leptomeninges possess anisotropic hysteretic abilities, like any biological elastic tissue [13]. The vessels and spaces that run through and cross the meninges, as well as the medullary and cranial spaces and lymphatic vessels, make the meninges a single functional unit [12,13]. The injury of a meningeal area will negatively affect the general function of the meninges themselves, and of the structures with which they come into contact [12-14]. According to the FNC and FORCE, the meninges are classified as fascial tissue, despite having a dual embryological derivation: mesoderm and ectoderm (Figure 1).

Fascial tissue embryology: bone tissue

Likewise, bone tissue has a dual phylogeny. Bone development involves the mesodermal sheet and the ectodermal sheet, whereas some bones derive only from the mesoderm (for example, the parietal bone), some derive from the contribution of both sheets (for example the frontal bone), and some arise directly from the ectoderm like the maxillary bone [12-17]. The speech is more complex, as many bone progenitor cells interact with other structures that will transform into non-bone tissues, such as the neural tube and dura mater; defining an exact boundary between the two embryological sheets and classifying the tissues deriving from each origin, without taking into account the ontogenetic mixture, it can be revealed an error [18]. Furthermore, some mesodermal and ectodermal cells share the same characteristics and it is not easy to separate their origin; again, some bone sutures, for example between the two parietal bones fuse like a strip of ectodermal cells [19].

**Figure 2:**
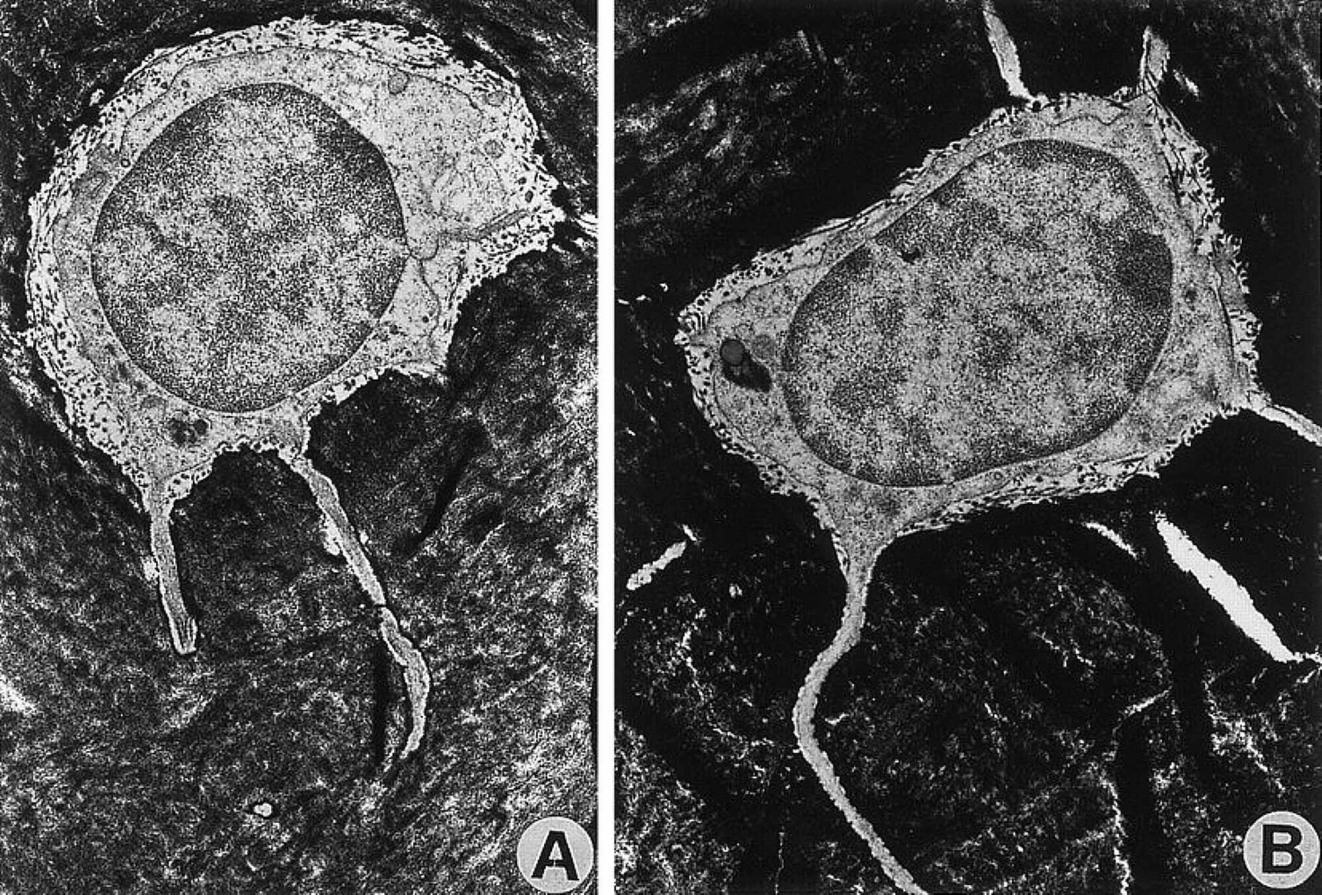
The electron microscope figure shows osteocytes within the cortex of different bones in the metatarsal area: (A) bone subjected to mechanical load; (B) bone without mechanical load. The presence or absence of load does not alter the microscopic structure of the osteocyte. Source: Image reproduced with permission of A. Rubinaci, MD, PhD, Bone Metabolic Unit, Scientific Institute H San Raffaele, 20132 Milano, Italy.

The morphological epigenetics of bone formation and muscle districts demonstrates how, during development, the size of a bone affects the size of the muscles and vice versa; if the bone tissue increases, the size of the muscles that will attach in the same area decreases and vice versa, if the size of the muscles increases, the size of the future bone tissue will decrease [20]. Bone tissue and musculature interact during development and during adult life, as they secrete hormones and growth factors that influence behavior in a reciprocal way, through autocrine and paracrine actions [16]. To give an example, depending on the type of muscle contraction, a greater quantity of myostatin will be produced (eccentric contraction); if there is an increase in synthesized myostatin, as with advancing age and the presence of sarcopenia, this growth factor will have an anti-osteogenic effect with a worsening of various clinical pictures [21-23]. According to FORCE, bone tissue is considered as fascial tissue, thanks to the double embryological derivation [8]. As with the meninges, where each area affects nearby and distant meningeal areas through mechanical tension, felt and produced, so the cells that make up the bone tissue are aware of what happens in the area where they are located and in distant areas of the bone. when there is a mechanical variation (Figure 2) [16,24].

Fascial tissue embryology: myofascial tissue

If the bone tissue derives from the ectodermal and mesodermal sheets, through the orchestration of multiple biochemical factors and the intervention of skeletogenic cells and chondrocytes, the connective tissue that interpenetrates with the contractile tissue has the same double origin [25]. The connective tissue of the skull that merges with the voluntary striated musculature, as well as with the musculature that will form the lingual complex and the subhyoid area, derives from the ectoderm [8]. The musculature of the cervical spine, such as the trapezius muscle and the sternocleidomastoid, demonstrates a combination of ectodermal and mesodermal connective tissue [8]. Why exclude ectodermal origin? FORCE includes in the definition of fascial tissue voluntary and connective striated musculature, although not always with the same ontogenic origin [8]. The myofascial tissue (muscle and connective) have similar characteristics. The different proteins that make up the myofascial macrostructure, such as sarcolemma and sub-sarcolemmal proteins, inter-filaments (IFs), cellular and nuclear proteins, and connective-forming proteins (glycoproteins and proteoglycans) respond to mechanical tensions and are capable of carrying mechanical information from the outside to the inside of the cell and vice versa.

Dystroglycan (α and β), is a protein complex of the sarcolemma, capable of bi-directionally managing mechanical tensions (pressure, alteration of position and shape) so that the cell is able to obtain a physiological adaptation to mechano-metabolic variations of its interior and exterior (extracellular matrix); if this capacity fails, or its presence diminishes, the cell will no longer be able to adapt and survive, with the formation of muscular dystrophy [26]. The dystrophin or sub-sarcolemmal protein that penetrates the cytosol and the sarcolemma, essential for correctly amortizing the various mechano-metabolic alterations; when deficient or absent, Becker and Duchenne dystrophy develop, respectively [27].

One of the proteins that make up the IFs protein complex and that connect the sarcolemma to the cell membrane is desmin. It allows maintaining the shape and function of the muscle cell, without which there would be a contractile functional alteration and desminopathy [28,29]. Telethonin is one of the many sarcomeric proteins, capable of maintaining the structural integrity of the membrane and the cytoskeleton, as is the lamina, a nuclear membrane protein, capable of managing the mechanical tensions that reach it; in case of alteration of these proteins, muscular pathologies can occur, such as limb-girdle muscular dystrophy and laminopathy, respectively [30,31]. Type 1 fibrillin is a glycoprotein, essential for maintaining mechano-metabolic homeostasis and for managing the shape of the connective scaffold; its functional and percentage alteration will create pathologies, such as Marfan syndrome [32]. Syndecans are transmembrane proteins, defined as proteoglycans, they are ubiquitous structures; they play a fundamental role in regulating the development of motor neurons and their deficiency could cause motor disturbances [33].

When a limb moves, all these structures that make up the myofascial system (and not only) are activated and connected. Likewise, when a myofascial area is subjected to surgical, physiotherapeutic or osteopathic or chiropractic treatment, no one can state which microscopic component is not involved, since the macroscopic is the result of the aggregation and interpenetration of the microscopic. All proteins and other molecular compounds contribute to the final function of the myofascial system. When surgery is performed to transfer a tendon district to restore the function of a muscle, the microscopic and macroscopic components are damaged; the final function remains altered [34]. When an osteopathic or chiropractic treatment is performed on a myofascial area, everything that represents the worked structure improves; it is not possible to divide the purely contractile component from the purely elastic component [35,36]. A physiotherapeutic approach to reduce nociceptive afferents from a myofascial area in the presence of trigger points will affect both the contractile component and the elastic component [37].

Starting from the microscopic components, we can conclude that the macroscopic has no layers, but has absolute continuity. The collective imagination refers to the term layer as a two-dimensional structure but, in reality, the whole body is a three-dimensional complex, where cells interconnect directly and indirectly (electrical activity, hormones, biochemicals, mechanical tensions). To think that during an anatomical dissection or a surgery to remove a tissue means only removing some layers is equivalent to saying that the human body is a set of cells that form tissues that are not connected to each other. When a tissue is removed, the whole body is involved and must adapt, and all the systems directly connected to that tissue (nerve filaments, vascular and lymphatic pathways, extracellular fluids, cells found in multiple tissues) are negatively involved.

Fascial tissue embryology: smooth muscle and non-voluntary striated musculature

Solid fascial tissue is able to contract. An example is the cyclic contraction capacity of fibroblasts, ubiquitous structures, where their constant alteration produces a permanent contracture effect, transforming into myofibroblasts [38,39]. The possibility of contraction of the fascial tissue thanks to its fibroblastic components is a known notion since 1968 [40]. The smooth muscles of the gastrointestinal tract and the excretory system derive from the mesodermal sheet [41]. The mesoderm is divided into intermediate, paraxial, and lateral plate mesoderm; from the latter derives the smooth and striated muscle, as well as the involuntary striated muscle [41]. From the lateral plate of the mesoderm different components of the gastric system will derive in perfect synergy with the endodermal sheet; movement and visceral elasticity depend entirely on the mesoderm, thanks to fibroblasts and myofibroblasts for their contractile component (alpha and gamma-smooth muscle actin) [41].

During development, smooth muscle cells are essential for correct migration and location of the enteric system, while in the adult stage, smooth cells are important for the proper functioning of the enteric system [41]. Another mesodermal protein that is found in smooth muscle (and in all tissues) is the telocyte; it allows proper communication between intestinal electrical activity and muscle contraction [41]. The digestive system starts from the mouth and ends at the anus and we can find visceral muscle components in different structures, such as the pharynx and esophagus. The musculature of the pharynx is of the involuntary striated type and rich in elastic tissue; all the musculature that governs the pharynx derives from the mesoderm [41]. The esophagus that derives from the mesoderm has a portion of smooth muscle (inferiorly) and a portion of striated muscle (superiorly); in particular, the striated musculature derives from the lateral mesodermal plate [41]. The gallbladder derives from the mesoderm and is richly covered with smooth muscle (muscularis propria), as well as the bile duct (muscularis mucosae); thanks to the muscular elasticity, both structures are able to correctly manage the mechanical tensions they feel and adapt their structure [41]. A visceral manual treatment is able to improve digestive functions in patients undergoing chemotherapy for breast cancer, as well as improve function parameters in the area of lumbar pain, after a visceral abdominal treatment with a manual approach [42,43]. The manual approach has the same principles for a myofascial type treatment, and one could speak of viscerofascial [44]. The visceral mesoderm will supply the cartilaginous skeleton of the larynx (lamina propria) and the laryngeal vascular system; the involuntary striated musculature of the larynx, extrinsic and intrinsic, derives from the IV and VI mesodermal pharyngeal arch [41].

Continuing with the respiratory tract, the anterolateral portion of the tracheal cartilage structure, and the posterior area, derive from the mesoderm; the posterior area merges with the smooth muscle [41]. During the development of the trachea, the future tracheal musculature, through the synthesis of some growth factors, allows the endoderm to properly form the remaining internal part of the tracheal organ [41]. The smooth muscle of the trachea contracts rhythmically in the adult, creating a kind of peristalsis and thus allowing to stimulate the mechanostraductive processes and maintain the shape and function of the trachea [41]. The external cartilage portion of the bronchus and the smooth muscle derive from the mesoderm, just as the pleurae (visceral and parietal) originate from the mesoderm [41]. There are different types of receptors on the pleura and thanks to the intrinsic elastic capacity, they inform the central nervous system of the pulmonary expansive capacity, or if there is a pathogen that limits pulmonary movement; thanks to these afferents, the immune system and respiratory health are reflexively stimulated [41]. Although the manual approach to the respiratory system may not reveal evidence of certainty, the manual approach to the lung area is safe, with positive responses of some respiratory parameters (forced expired volume in 1 second - FEV1) and residual volume (RV) [45,46].

Several other visceral structures contain smooth muscle tissue with mesodermal derivation, such as the parenchyma of the spleen, the peritoneal membrane and the parietal peritoneum, including the visceral peritoneum; all the smooth muscles of the ureters, the striated (or external) sphincter muscle of the urethra, the fibrous capsule of the kidney (with smooth contractile fibers), the capsule of the adrenal gland, the muscles of the bladder and uterus derive from the mesodermal sheet [41]. Thanks to the presence of mesodermal derivation, these tissues adapt very quickly to mechano-metabolic stimuli, managing visceral function through the information they receive and send back [41]. According to some authors, the involuntary striated muscle or cardiac myofascial muscle derives from a fourth mesodermal sheet or cardiogenic mesoderm [47]. The cells and future cardiomyocytes will join the ectodermal cells (first cardiac field); during development, myocytes deriving from the mesoderm will orient themselves towards formation from cardiomyocytes or towards electrical conduction cells [47]. The mechanical information that the heart receives and sends, through varying blood pressures, the movement of the respiratory diaphragm, the pulmonary expansion, play a main role in the physiological continuation of its function; the heart is able to change shape and position according to the need or the mechanical tensions present [48]. The smooth, voluntary and involuntary contractile muscles, permeated by collagen, have multiple characteristics in common. For every breath, every movement of the body, and every heartbeat, all muscle cells, regardless of their phenotype, are subject to mechano-metabolic information [49]. And this concept starts from the microscopic, that is, from the molecular structures that make up cells.

Deformation of the cells also occurs without external mechanical perturbations, through known phenomena. Through the cytoskeletal polymerization of actin groups or individual protein filaments that actively push the membrane from the inside, or through hydrostatic forces within the cell that allow the same structures to swell the membrane; by doing so, the cells can probe the external environment, creating thin filaments or filopodia or they can invade the extracellular matrix through filaments called invadopodia [49]. The cell is able to withdraw these thin filaments through contractile forces, thanks to the type II myosin that flows on the actin; the information that returns to the cell allows the mechano-metabolic message, also through the depolymerization of microtubules and actin, to reach the cell nucleus and move chromosomes and stimulate cytokinesis [49]. Considering that the muscle fibers (smooth and striated) are interpenetrated in the different structures of the organ itself, are fused with collagen, or are adjacent to multiple structures (bone, visceral, muscular, nervous, vascular, and lymphatic), and are the same fibers which allow the distribution of the mechanical and then metabolic tension felt or created, we can consider the contractile fibers as fascia [50].

## Conclusions

The article reviewed concepts related to the fascial system and the most up-to-date research and articles to give a definition and nomenclature of what could be considered the fascial continuum, emphasizing some clinical and pathological connotations. The text illustrated some embryological notions, as the authors believe that starting from the knowledge of the origin of the tissues, it is possible to identify the most correct connotation of the same tissue. The continuous renewal of scientific research makes it possible to observe knowledge through further points of view; different opinions coming from different scientific figures are essential to better frame a topic. Fascial tissue is a topic available to everyone and not a market where only an elite of a specific health specialization can express an opinion. In the second part of the article, we will highlight a definition of what could be considered the fascial continuum, through the multidisciplinary experience of FORCE.
